# Arsenic trioxide as a novel anti-glioma drug: a review

**DOI:** 10.1186/s11658-020-00236-7

**Published:** 2020-09-24

**Authors:** Yi Fang, Zhen Zhang

**Affiliations:** Department of Ultrasound, First Affiliated Hospital of China Medical University, Shenyang, 110001 Liaoning People’s Republic of China

**Keywords:** Arsenic trioxide, Glioma, Anti-cancer mechanism

## Abstract

Arsenic trioxide has shown a strong anti-tumor effect with little toxicity when used in the treatment of acute promyelocytic leukemia (APL). An effect on glioma has also been shown. Its mechanisms include regulation of apoptosis and autophagy; promotion of the intracellular production of reactive oxygen species, causing oxidative damage; and inhibition of tumor stem cells. However, glioma cells and tissues from other sources show different responses to arsenic trioxide. Researchers are working to enhance its efficacy in anti-glioma treatments and reducing any adverse reactions. Here, we review recent research on the efficacy and mechanisms of action of arsenic trioxide in the treatment of gliomas to provide guidance for future studies.

## Introduction

Glioma is the most common primary malignant tumor of the central nervous system, accounting for about 50–60% of intracranial tumors. Its invasion and recurrence rates are significantly higher than those of other intracranial tumors [1]. Due to its invasive growth, it generally cannot be completely surgically removed [2]. Despite advances in clinical treatment methods, the prognosis for patients remains poor [3]. New therapeutic strategies, including novel drugs, are needed to effectively inhibit the proliferation, invasion and metastasis of glioma cells and treat this malignant cancer type.

Elemental arsenic is insoluble in water and acid. It has almost no toxicity, but when exposed to air, it readily oxidizes to highly toxic arsenic trioxide. Traditional Chinese medicine has applied arsenic and arsenic-containing medicines to treat a variety of intractable diseases since ancient times [4], but the toxicity has limited its medical applications. Recent studies have shown that arsenic trioxide has an inhibitory effect on acute promyelocytic leukemia (APL), a hematological tumor, and on solid tumors including liver [5], lung [6] and breast cancer [7].

In glioma, arsenic trioxide exerts its anti-cancer effects via regulation of apoptosis and autophagy; its impact on the cell cycle; promotion of the production of intracellular reactive oxygen species (ROS), which results in oxidative damage; inhibition of tumor stem cells; and enhancement of the effects of radiotherapy and chemotherapy. However, glioma cells and tissues from various sources have different responses to arsenic trioxide, and it has some limitations and side effects in tumor therapy [8–10]. Research in this area is increasingly focused on combinations of arsenic trioxide with other drugs or methods to enhance its anti-glioma efficacy and reduce adverse reactions.

## Arsenic trioxide induces programmed cell death

There are two forms of programmed cell death, apoptosis and autophagy [11, 12]. Apoptosis is governed by two common pathways: the extrinsic path way initiated by the binding of death ligands to death receptors on the plasma membrane; and the intrinsic pathway, where cellular oxidative stress leads to a loss of mitochondrial integrity and their destruction [13]. Both pathways activate caspase-3.

Autophagy is characterized by the formation of acidic vesicular organelles in the cytoplasm [14]. This causes the destruction of the nucleus and the collapse of cytoplasmic or ganelles. Autophagy can prevent toxic accumulation of cell waste, protect organelles and maintain cell survival. It has also been shown to promote the development of tumor cells and has been implicated in chemotherapy resistance, although it can also inhibit tumor growth in some cases [15].

Similar stimuli can induce apoptosis, autophagy or both, possibly because apoptosis and autophagy have common effector proteins (including Bcl-2, Bcl-xl, Mcl-1, ATG5 and p53) and upstream pathways (including PI3K/Akt/mTOR, NF-κB and MAPK) [16]. Therefore, apoptosis and autophagy may be related or even regulated by the same trigger in the anti-glioma mechanism of arsenic trioxide [17, 18].

In experiments with six malignant glioma cell lines (U373, U87MG, U251, GB1, A-172 and T98G), Kanzaw et al*.* showed that treatment with a low concentration of arsenic trioxide (2 μM) caused glioma cell death but could not induce apoptosis. U373 cells showed the loss of microvilli and the presence of a large number of autophagic vacuoles. The same researchers studied the occurrence of autophagy and found that arsenic trioxide upregulated mitochondrial cell death protein BNIP3, thereby opening mitochondrial permeability transition pores and destroying the integrity of the mitochondrial membrane [19].An inhibitor of caspase-3 could not prevent cell death induced by arsenic trioxide, but apoptosis occurred when the autophagy inhibitor bafilomycin A1 was used in combination with arsenic trioxide [20]. Therefore, at low concentrations of arsenic trioxide, if autophagy is inhibited, apoptosis occurs as an alternate pathway.

Similar studies have also shown that inhibiting cathepsin L (CatL) activity partially reduces lysosomal activity, thus affecting autophagy. This can also cause the transition from autophagy to apoptosis in U87MG cells that were treated with a low concentration of arsenic trioxide (2 μM).The drug effect is more obvious in multicellular spheroids than in cell monolayers [21].

The effects of arsenic trioxide on apoptosis and autophagy in glioma may be affected by the drug concentration and the p53 gene type. As arsenic trioxide-induced apoptosis in glioma requires complete p53 protein function, autophagy may become the dominant form of programmed death when apoptosis is inhibited [22]. In a wild-type p53 U87MG cell line, arsenic trioxide at concentrations higher than 4 μM was shown to induce apoptosis [23, 24]. It could also significantly induce the autophagic death of p53-mutant U118 cells. However, even at a high concentration (50 μM), it could not induce apoptosis or cause the activation of aspases-3, -8 and -9. Blocking the apoptosis pathway mediated by p53 may thus be one of the mechanisms by which autophagy is induced by arsenic trioxide [25, 26].

Further study showed that the induction of autophagy in glioma cells may also be related to the heat shock response. After treatment of U118 with arsenic trioxide, the expression of the heat shock protein HSP70 increased. In addition, the HSP70-family protein HSC73 bound to soluble proteins and initiated their transport to lysosomes or lysosomal compartments, resulting in complete protein degradation [27].

However,other reports have been inconsistent with the above-mentioned results (Table 1). For example, Chiu et al*.* found that arsenic trioxide had both apoptotic and autophagic effects on U118 cells, related to the inhibition of survivin [28]. Zhao et al*.* studied the expression profiles of apoptosis- and cell cycle-related genes in two glioma cell lines (U87MG and T98G) of different p53 types treated with arsenic trioxide (2 μM). The results suggested that arsenic trioxide inhibited the growth of U87MG cells expressing wild-type p53 mainly by affecting the expression of genes involved in cell cycle arrest, stress and toxicity. Furthermore, it inhibited the growth of p53-mutant T98G cells mainly by upregulating the apoptosis pathway mediated by Bcl-2 and tumor necrosis factor. They also found that arsenic trioxide was more likely to cause apoptosis of p53-mutant cells [29]. A Separate study have shown that 2 μM arsenic trioxide can induce apoptosis in U87MG, U251, SHG44 and C6 glioma cells in a dose-dependent manner [30].

**Table 1:** Studies on arsenic trioxide-induced glioma cell death

First author	Cell line	Does (μM)	Time	Findings	Refs
Kanzawa	U373, U87MG, U251, GB1, A-172, T98G	1,2,4	72 h	Autophagy	[19, 20]
Eun Hee Kim	U373MG, U87MG, U251MG, U343, U251N, T98G	4,5,6	24 h	No apoptosis	[30]
Ye Cheng	U87MG, U251, SHG44, C6	2,4,8	48 h	Apoptosis	[17]
Naomi Haga	A172, T98G	1,10,50	48 h	A172: apoptosis; T98G: no apoptosis	[40]
Yuanyuan Sun	C6, 9L	0.5–8	24,48,72 h	Apoptosis	[41]
Primon, M	U87MG	2,5,10,20	72 h	2 μM:autophagy; More than 10 μM: apoptosis	[21]
Karsy, M	U87MG	0.5,1,2,4,16	24 h	More than 4 μM: apoptosis	[23]
Majid Zaki Dizaji	U87MG	1,2	48 h	Apoptosis	[63]
G.-B. WANG	U87MG	4,6,8	72 h	Apoptosis	[24]
Shiguang Zhao	U87MG,T98G	1,2,4,8	12,24,48 h	Apoptosis, the effect of T98 is greater than U87	[28]
Hui-Wen Chiu	U118MG	2,4,6,8	12,18,24,36 h	Autophagy and apoptosis	[25]
Tain-Junn Cheng	U118	1,5,10,25,50	24 h	Autophagy	[24]
Hui-Wen Chiu	U118-MG	2	24 h	Autophagy	[27]
Yuanyan Wei	SHG44	2,4,6	24 h	Apoptosis	[36]

In conclusion, further study is needed to determine whether apoptosis or autophagy of glioma cells is induced by arsenic trioxide. However, regardless of the p53 type of gliomas, low-dose arsenic trioxide (1–2 μM) can induce programmed cell death in vitro, and it is effective as a single or combined therapy for glioma [23].

There are other mechanisms by which arsenic trioxide regulates programmed cell death in glioma cells. Some studies have found that 5 μM arsenic trioxide can upregulate the expression of the death receptor DR5 and facilitate its binding to tumor necrosis factor-related apoptotic ligand (TRAIL). This leads to both the cleavage of pro-caspase-3 (the precursor of caspase-3) and apoptosis via extrinsic pathways. The upregulation of DR5 induced by arsenic trioxide may be mediated by CCAAT/enhancer-binding protein homologous protein (CHOP) [31]. CHOP is stimulated by DNA damage and is one of the most strongly induced genes during endoplasmic reticulum stress. However, no DNA damage was detected in the cited study, possibly due to the low dose of arsenic trioxide, which is known to have no cytotoxic effect on human normal glial cells. This could be attributed to the low expression of DR4 and DR5 in normal glial cells, which would make them insensitive to apoptosis induced by TRAIL [32].

Apoptosis is also thought to be related to glycosylation. The sugar chain structure of cell surface proteins has an important role in regulating cell survival [33–35]. Arsenic trioxide can affect apoptosis by reducing the expression of a member of the β1,4-galactosyltransferase family: β1,4 GalT V, which galactosylates the β1,6-GlcNAc branch of N-glycans [36, 37].

**Fig. 1:**
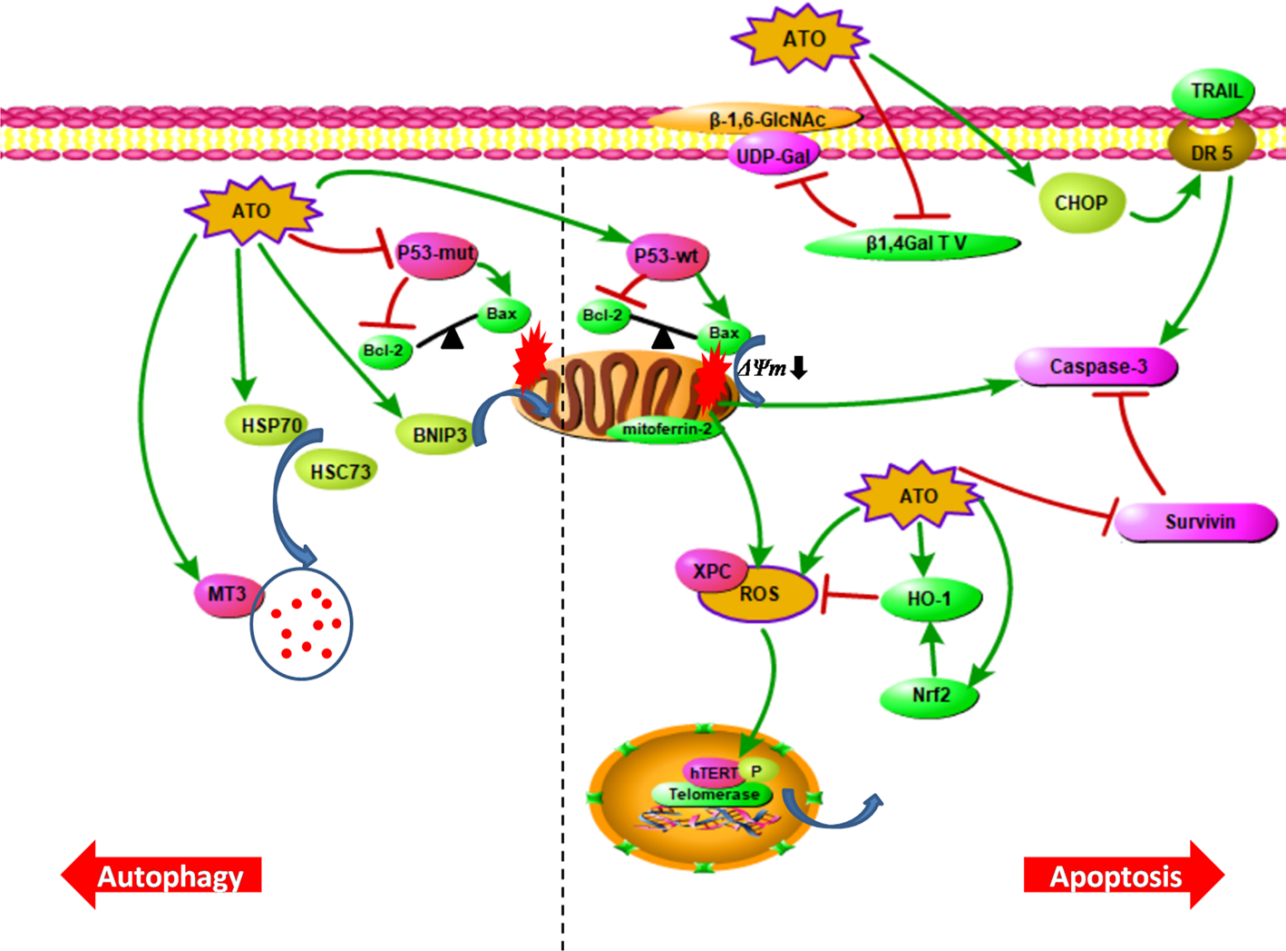
The mechanisms of glioma cell death induced by arsenic trioxide. Apoptosis can be induced via external pathways and through internal pathways in glioma cell lines containing wild-type p53. Arsenic trioxide can also cause cell death in glioma through autophagy

In cells treated with arsenic trioxide (6 μM) for 48 h, about 40% of the water-soluble arsenic present was found to exist in the metallothionein (MT) protein component. MTs can bind to metal elements, thereby protecting cells from arsenic toxicity. Further studies of the interactions between arsenic trioxide and MT subtypes, with different doses or durations of treatment, indicated that the expression of the MT gene in each subtype increased or decreased to a varying degree. The mRNA expression levels of MT1X, MT1F and MT2A increased the most (up to 13 times), whereas those of MT1A, MT1E and MT3 decreased or remained unchanged. The increase in expression of MT1X, MT1F and MT2A may be one of the mechanisms by which glioma cells resist the toxicity caused by arsenic trioxide therapy. On the other hand, MT3 has low metal inducibility and is capable of rapid binding and release of metals. Its isoform is mainly expressed in the brain and it is necessary for the normal lysosomal function of astrocytes. In astrocytes with MT3 deletion, the activities of several lysosomal enzymes decreased significantly, and the death of glioma cells induced by oxidative stress was substantially weakened [38]. After treatment with arsenic trioxide (7 μM) for 24 h, MT3 expression increased. Therefore, the involvement of MT3 in autophagic cell death induced by arsenic trioxide cannot be ruled out (Fig. 1) [39].

## Arsenic trioxide acts through oxidative damage

Compared with other organs, the brain seems to be particularly sensitive to ROS stress. Although it accounts for only 2% of bodyweight, it consumes up to 20% of the body’s oxygen supply. Large amounts of ROS are produced in the brain tissue during oxidative phosphorylation [40]. Since tumor cells are very sensitive to the stress response to ROS, treatments that affect ROS levels may also affect tumors.

The mechanisms by which arsenic trioxide induces apoptosis include stimulate on of ROS production, mitochondrial aggregation, Bax oligomerization, dissipation of membrane potential, and mitochondrial membrane collapse, followed by the release of apoptotic factors, the caspase cascade, and, finally, cell death. Increased ROS levels were detected in both A172 and T98G cell lines treated with 50 μM arsenic trioxide. However, there was no mitochondrial aggregation, membrane potential dissipation or Bax oligomerization in these T98G cells. The mitochondria of T98G cells may be less sensitive and responsive to oxidative stress, and thus resistant to arsenic trioxide–induced mitochondrially mediated apoptosis [41].

A study using rat C6 and 9L cell lines showed that 5 μM arsenic trioxide could strongly inhibit cell viability and induce apoptosis by downregulating the expression of Bcl-2 and upregulating the expression of Bax. This change is related to the mitochondrial damage caused by the production of intracellular ROS. However, the inhibition rate for normal rat glial cells was less than 10% of that for rat glioma cells [42].

While arsenic trioxide increased the production of ROS in glioma cells, it also increased the expression of heme oxygenase-1 (HO-1) and its upstream effector Nrf2. HO-1 can protect against oxidative stress and antagonize the effects of ROS. Therefore, inhibiting the expression of the gene encoding HO-1 or Nrf2 can enhance the oxidative damage caused by arsenic trioxide [43].

Iron plays an important part in the production of ROS by mitochondria [44]. Mitoferrin-2 is a mitochondrial protein that mediates ferrous transport through the mitochondrial inner membrane. The production of excess mitochondrial ROS requires more iron to pass through the mitochondrial membrane. In U87MG and T98G cells treated with arsenic trioxide, expression levels of mitoferrin-2 increased four- to fivefold, while ROS production and apoptosis decreased in the cells with low expression of mitoferrin-2. Silencing mitoferrin-2 led to a decrease in arsenic trioxide cytotoxicity. Therefore, the mitoferrin-2 transporter could participate in arsenic trioxide-induced cytotoxicity by promoting the production of ROS [45].

Other studies have shown that arsenic trioxide could induce the expression of XPC (xeroderma pigmentosum, group C) in U87 cells. XPC may be involved in the regulation of the intracellular redox dynamic balance. When it was silenced, U87 cells were more sensitive to arsenic trioxide [46].

Telomeres are a special DNA structure located at the ends of chromosomes, which are gradually shortened with each cell division. It enables cells to avoid proliferative disorder by stabilizing the telomeres, especially through overexpression of its catalytic subunit hTERT, which is found in 85% of cancers, including glioblastoma [47]. Arsenic trioxide treatment causes DNA double-strand breaks due to the related increase in intracellular ROS production. This induces phosphorylation of hTERT and its subsequent translocation from the nucleus to the cytoplasm, and dysfunction of telomerase function, resulting in apoptosis, cell cycle arrest and other cellular changes (Fig. 1) [30].

## Arsenic trioxide causes G1 or G2/M phase arrest

In most solid tumor cells, arsenic trioxide induces G1 or G2/M phase arrest of the cell cycle [48]. Cell cycle arrest before mitosis occurs in response to DNA damage, enabling cells to repair damage and preventing genetic errors from being transmitted to daughter cells [49]. It may be the main response to such damage, occurring before the decision to repair or die. In glioma cells treated with a low concentration of arsenic trioxide (2 μM), the cell cycle was blocked in G2/M phase [20], and the levels of cyclinB1, aurora kinase A, and phosphorylated aurora kinase A decreased in a dose-dependent manner. There was no significant change in the levels of cyclinD1, indicating no G1/S phase block [30]. This may be because arsenic trioxide increases the expression of wild-type p53, which can inhibit the cdc25C promoter, which in turn inhibits the activation of cyclinB1, resulting in G2/M phase arrest [50].

## The influence of arsenic trioxide on microRNAs (miRNAs)

The anti-cancer effects of arsenic trioxide may be related at least in part to epigenetic regulation of miRNAs. It may alter the expression levels of a large number of miRNAs with important roles in cancer–related signaling pathways by affecting DNA demethylation [51]. Many of these miRNAs are considered to be tumor suppressor genes or oncogenes.

Shidfar et al*.* treated U87 cells with 4 μM arsenic trioxide for 48 h and applied gene chip technology to analyze the changes inexpression of 88 cancer-related miRNAs. They found that 60 miRNAs showed increased expression and 28 showed decreased expression [52]. Nine miRNAs were significantly upregulated (i.e., a greater than four-fold increase in expression). The most strongly upregulated miRNAs were miR-215 and miR-96, with differential expression rates of about 13- and ninefold, respectively. In addition, a fourfold decrease in expression levels was detected for three miRNAs. Overall, the upregulation of miRNA was more significant, and more were upregulated than downregulated. A large proportion of the significantly upregulated miRNAs (including miR–215, -96, -126, -149, -193a-5p and -183) had inhibitory effects on tumors and metastasis. However, of the significantly downregulated miRNAs, only two (miR-27b and miR-100) have reported carcinogenic effects.

In a separate study, Wang et al*.* treated U251 cells with arsenic trioxide and showed that it directly inhibited potassium hERG channels at a post-transcriptional level by upregulating miR-133b, thereby causing cell apoptosis [53].

## Inhibitory effects of arsenic trioxide on glioma stem cells

To some extent, the recurrence of glioblastoma is mediated by glioblastoma stem cells (GSCs). Arsenic trioxide can reduce the expression of stem cell markers (SOX2, CD133) of GSCs and inhibit the Hedgehog and Notch pathways, thus reducing their sphere forming ability, inhibiting their DNA repair ability, and reducing drug resistance [54]. A combination with (–)-gossypol can enhance this inhibitory effect [55].

Bureta et al*. *compared the effects of temozolomide combined either with arsenic trioxide or with the Hedgehog pathway inhibitor vismodegib. Both combinations increased the anti-glioma effect of temozolomide, with similar effects, and inhibited the Hedgehog pathway in glioblastoma [56].

When U87MG, U251MG and U373MG GSC cells were treated with arsenic trioxide, the protein levels of Notch1 and Hes1 decreased significantly, indicating that it inhibited the survival and proliferation of tumor stem cells by blocking the activity of the Notch signaling pathway [57]. A further study targeting the Notch pathway showed that treatment with 2 μM arsenic trioxide initially hindered the proliferation of neurospheres, after which the cells could recover. However, treatment with 4 μM arsenic trioxide reduced the recovery of neurosphere formation ability and significantly reduced the formation of tumors. In addition, when the remaining neurospheres were isolated and replicated, it was found that the cells treated with 4 μM arsenic trioxide no longer had the ability to renew themselves because they could not form secondary neurospheres. This phenomenon was related to the inhibition of phosphorylation and activation of AKT and STAT3 via the blocking of Notch signaling [58].

Currently, promyelocytic leukemia (PML) protein is the main target in the treatment of APL, which is the most common use of arsenic trioxide [59, 60]. In vivo and in vitro experiments showed that arsenic trioxide can reduce the expression of PML in GSCs, leading to the ubiquity in degradation of c-Myc and thus inhibiting GSCs [61].

GBM can be divided into four molecular subtypes: proneuronal, neural, classical and mesenchymal. GSCs are key promoters of the occurrence and evolution of GBM and lead to drug resistance and recurrence of tumors. The mesenchymal subtype is more invasive and has a poorer prognosis than the proneuronal subtype [62]. Bell et al. proposed that the different subtypes of GSC have different responses to arsenic trioxide, with a better therapeutic effect in proneuronal than in mesenchymal. This may be related to the overexpression of many oncogenes caused by the activation of MNK1-eIF4E signaling in the mesenchymal subtype. When arsenic trioxide and a signaling pathway inhibitor were used together, the drug resistance of mesenchymal glioblastoma could be reversed [63].

## Enhanced anti-glioma effect of arsenic trioxide combined with other drugs or methods

Combined treatment strategies have become the most common approach to overcoming malignant tumor resistance to anti-cancer drugs. Arsenic trioxide has potential in combined strategies but its toxicity and side effects are an issue.

A combination of arsenic trioxide and silibinin was shown to synergistically inhibit U87 metabolic activity, cell proliferation, and gelatinase A and B activities; increase cell apoptosis; decrease the mRNA levels of cathepsin B, urokinase type plasminogen activator, matrix metallopeptidase 2 (MMP-2), MMP-9, survivin, Bcl2 and CA9; and upregulate caspase-3 mRNA expression [64]. The mechanism of the sensitization of cells to arsenic trioxide by chrysin and silybin was studied using the A172 cell line. The results showed that chrysin enhanced the cytotoxicity of arsenic trioxide by inducing glutathione (GSH) depletion and promoting arsenic accumulation.

GSH is the most important intracellular antioxidant. Its depletion can increase the toxicity of arsenic in various cell types via mechanisms including increased oxidative stress and free active arsenic concentration, loss of arsenic-bound mercaptan, and reduction in the detoxification by GSH–dependent methylation and the binding of arsenic to GSH. Silibinin sensitization did not affect GSH levels but doubled the accumulation of arsenic. Accumulation of arsenic may be related to the inhibition of the ATP-binding cassette transporter and multidrug resistance-associated protein, which would slow down the excretion of arsenic and increase its accumulation [65]. It was also found that butylthionine sulfoxide, an inhibitor of GSH biosynthesis, could deplete intracellular GSH and had a synergistic effect with arsenic trioxide in the C6 cell line [66].

Lin et al. found that berberine could enhance the arsenic trioxide-mediated inhibition of the migration and invasiveness of glioma by reducing the activation of protein kinase C signaling and MMP-2 in the extracellular matrix [67].

Mesbahi et al. found that the most common genetic change in malignant glioma was the amplification of epidermal growth factor receptors (EGFR, ERRB1 or HER1). Arsenic trioxide treatment alone could induce the activation of the EGFR signaling pathway in U87MG and A172 cells. Over activation of this pathway may be directly related to resistance to arsenic trioxide as a drug. When erlotinib, an inhibitor of EGFR, was used in combination with arsenic trioxide, ROS levels increased and there were significant synergistic effects on survival, proliferation and migration as well as on the cell cycle [68]. The activation of downstream products of EGFR is related to the regulation of c-Myc. A combination of arsenic trioxide and c-Myc inhibitors can also inhibit glioma [69].

Many researchers have also studied the combined effects of arsenic trioxide and radiotherapy. Ning et al. treated U87MG and SNB75 cells with a combination of radiotherapy and arsenic trioxide in vivo and in vitro. The results showed that arsenic trioxide sensitized glioma to radiation, with the greatest effect when it was administered 4 h after radiotherapy. However, the effects of arsenic trioxide alone or before radiotherapy were limited and this form of radio sensitization was time and sequence dependent [70, 71]. This may be because pre-administration leads to vascular closure, which lasts long enough for the tumor cells to produce a compensatory antioxidant response to both arsenic trioxide and the ROS produced by radiotherapy. By contrast, post-radiation administration of arsenic trioxide as a maintenance therapy may induce further damage through ROS and potentially reduce the ability of the tumor cells to repair the radiation-induced damage [72, 73].

A study of the influence of 2 μM arsenic trioxide and 4 Gy irradiation on U118-MG cells found that the proportion of mitotic cells increased significantly. Arsenic trioxide may inhibit the activation of G2-phase DNA damage checkpoints, thereby allowing cells with DNA damage to enter mitosis from the G2 phase. Entering mitosis in the presence of DNA damage leads to centro some inactivation and obstruction of chromosome separation, delaying the mitotic process and inducing mitotic abnormalities. This could lead to mitotic arrest and subsequent autophagy in malignant glioma cells. This combination could also induce autophagy of U118–MG cells by inhibiting PI3K/Akt and activating ERK1/2 signaling pathways [26].

## A clinical trial of arsenic trioxide in glioma treatment

Before 2000, clinical trials of arsenic trioxide focused on the treatment of APL. After treatment with a dose of 0.15 mg/kg/day for 12 to 39 days, most patients with APL achieved complete remission [74]. After its approval as a chemotherapeutic drug for APL by the U.S. Food and Drug Administration in 2000, trials were carried out to determine the clinical effects of arsenic trioxide on various solid tumors, including liver cancer [75], renal cell carcinoma [76], germ cell tumors [77], urothelial tumors [78], pancreatic cancer [79], breast cancer [80], head and neck cancers [81], and colon cancer [82, 83]. Based on the results, arsenic trioxide alone was not recommended as a chemotherapeutic drug for solid tumors.

Phase I and II clinical trials of glioma therapies were conducted at Northwestern University in Chicago to select the best combination dosage of arsenic trioxide, temozolomide and radiotherapy. When arsenic trioxide (0.2 mg/kg) was added to the standard glioma treatment regimen of temozolomide combined with chemotherapy, the survival rate of patients with anaplastic astrocytoma increased, but there was no significant improvement in patients with malignant glioblastoma [84, 85]. This may have been related to the presence of the blood–tumor barrier and the blood–brain barrier (BBB). Although there is evidence that gliomas involve partial BBB rupture or that a small amount of arsenic trioxide can pass through the BBB, this has not been shown to be sufficient for a curative effect. The low permeability of the BBB to drugs, including arsenic trioxide greatly limits the treatment of glioma. Increasing the ability of arsenic trioxide to pass through the BBB is key to improving clinical treatment.

## Transmittance of arsenic trioxide to the BBB

In the in vitro BBB model constructed by Tao et al., the transport ratio of arsenic trioxide was 2.21 ± 0.19%. In vivo, the elimination half-life of arsenic trioxide at the end of treatment was 4.41 ± 0.87 h, and the area under the drug-time curve was 0.18 ± 0.03 μg h/ml. After 24 h of intravenous injection, the arsenic trioxide uptake rate in glioma was 0.8 ± 0.3% ID/g, and the mean residence time was 6.17 ± 1.29 h. In this study, polyacrylic acid (PAA) was grafted onto mesoporous silica nanoparticle (MSN) cores to form MSN lipid capsule nanoparticles modified by self-assembled angiopep-2 (amino acid residues TFFYGGSRGKRNNFKTEEY). These could be loaded with arsenic trioxide (ANG-LP-PAA-MSN@As2O3).Owing to the specific recognition and binding of the BBB by angiopep-2, and the high expression of low-density lipoprotein related receptor 1 in glioma cells, the ability of arsenic trioxide to cross the BBB and target glioma increased, and pH-sensitive release [86]. The same research group used polyamidoamine (PAMAM) as a nano-carrier, which was connected to arginine glycine aspartic acid (RGD) for tumor targeting and loaded with arsenic trioxide to form RGDyC mPEG PAMAM/arsenic trioxide. Therapeutic drug delivery could also be achieved by combining arsenic trioxide with integrin αvβ3 [87].

## Conclusions

Although arsenic trioxide exerts anti-glioma effects through a variety of mechanisms, multidrug resistance and the existence of the BBB render glioma insensitive to it. In addition, serious adverse reactions to high dose arsenic trioxide limit its clinical applications. Therefore, it is necessary to further study the efficacy on glioma of a combination of drugs with arsenic trioxide as well as the efficacy of drugs encapsulated with arsenic trioxide carriers.

In addition, prior to preclinical trials and clinical application, precision medicine principles should be used to guide experimental research. It is essential that we search for tissue markers of glioma that are sensitive to arsenic trioxide. Finally, most studies to date have been conducted in vitro. Therefore, more preclinical and clinical studies should be conducted to elucidate the anti-cancer effects of arsenic trioxide in the treatment of glioma.

## Data Availability

Not applicable.

## Abbreviations

APL: Acute promyelocytic leukemia
BBB: Bood–brain barrier
CHOP: CCAAT/enhancer binding protein homologous protein
EGFR: Epidermal growth factor receptor
GSH: Glutathione
GSCs: Glioblastoma stem cells
HO-1: Heme oxygenase-1
MMP: Matrix metallopeptidase
MSN: Mesoporous silica nanoparticles
MT: Metallothionein
PAA: Polyacrylic acid
PAMAM: Polyamidoamine
RGD: Arginine–glycine–aspartic acid
ROS: Reactive oxygen species
TRAIL: Tumor necrosis factor-related apoptotic ligand
XPC: Xeroderma pigmentosum, group C
